# Influence of Total Anthocyanins from Bitter Melon (*Momordica charantia *Linn.) as Antidiabetic and Radical Scavenging Agents

**Published:** 2016

**Authors:** Aytaç Güdr

**Affiliations:** *Vocational High School of Health Services, Department of Medical Services and Techniques, Giresun University, Giresun, Turkey.*

**Keywords:** Antidiabetic activity, radical scavenging activity, total anthocyanin, bitter melon, harvesting time

## Abstract

The majority of the antioxidant and antidiabetic activities of fruits are anthocyanins; a group of polyphenolics that are responsible for the color of many fruits, vegetables and flowers. The harvesting time, storage conditions, maturity, extraction steps *etc*. are very important for the biological activities based on the alteration of chemical composition. The free radical scavenging and antidiabetic activities of total anthocyanins from bitter melon (*Momordica charantia* Linn) fruit (TAMC) were evaluated by considering four harvesting times. The free radical scavenging activities of the TAMC samples were assessed using DPPH^•^, DMPD^•+^ and ABTS^•+^ assays against BHA, rutin and trolox standards. September as a harvesting period (TAMC-S) had effective DPPH^•^ (SC_50 _2.55 ± 0.08 μg/mL), DMPD^•+^ (SC_50 _2.68 ± 0.09 μg/mL) and ABTS^•+^ (SC_50 _8.19 ± 0.09 μg/mL) scavenging activities compared with other samples and standards. In addition, August (TAMC-A) as a harvesting period showed very influential inhibitory activity against α-amylase (IC_50_ 56.86 ± 1.12 μg/mL) and moderate inhibitory activity against α-glucosidase (IC_50_ 88.19 ± 0.74 μg/mL). In comparison, pharmaceutical active ingredients such as acarbose exhibited anti-amylase and anti-glucosidase activities with IC_50_ values of 93.07 ± 1.49 μg/mL and 77.25 ± 1.20 μg/mL respectively. These results suggest that the correct selection of harvest period can significantly increase anthocyanin quantity because of the pharmaceutic properties of TAMC. Consequently, TAMC may be interesting for incorporation in pharmaceutical preparations for human health, since it can suppress hyperglycaemia that can be also used as food additives due to its antiradical activity.

## Introduction

The anthocyanins constitute a main flavonoid group that is responsible for cyanic colors ranging from salmon pink, red and violet to dark blue most of flowers, fruits and leaves of angiosperms (1). There has been an explosive interest in anthocyanins as potential nutritional supplements for humans. The regular consumption of anthocyanins in diet from fruits, vegetables, wines, jams and preserves is associated with probable reduced risks of chronic diseases *i.e*. cancer, coronary heart disease, diabetes mellitus, hypertension, cataract, virus inhibition and Alzheimer’s disease. Anthocyanins are regarded as important nutraceuticals because of their antioxidant effects, which give them a potential role in prevention of the various diseases associated with oxidative stress (2, 3).

Reactive oxygen species (ROS), which comprise free radicals like superoxide anion radicals (O_2_^•-^), hydroxyl radicals (HO^•^) and non-free radical species such as H_2_O_2_ and singlet oxygen (^1^O_2_), are form of activated oxygen (4, 5). ROS, which are produced *in-vivo* continuously, result in cell death and tissue damage. The role of oxygen radicals has been implicated in aging and several diseases for example arteriosclerosis, diabetes, cancer and cirrhosis *etc*. (5, 6). The various ROS in living organisms can be formed by different ways. These ways are classified as endogenous and exogenous sources (7, 8). The free radical scavenging activity of antioxidants in foods has been substantially investigated and reported in the literature. Many antioxidant compounds, naturally occurring from plant sources, have been identified as free radical or active oxygen scavengers (9, 10).

Both types of diabetes mellitus which are type I insulin dependent diabetes mellitus (IDDM) and type II noninsulin-dependent diabetes mellitus (NIDDM) are common and serious metabolic disorders throughout the world. Traditional plant treatments have been used in the world for the therapy of diabetes mellitus. Among many medications and other alternative medicines, several herbs have been known to cure and control diabetes; additionally they have no side effects. Plant-based medicine has been used cost-effectively worldwide to treat diabetes mellitus. In fact, in many parts of the world, this may be the only form of therapy available to treat diabetic patients. There are several reviews by different authors about anti-diabetic herbal plants (11, 12).

*Momordica charantia* Linn. (Cucurbitaceae) commonly known as bitter melon, balsam apple, bitter gourd and bitter squash is a multi-purpose herb widely cultivated in many tropical and subtropical regions of the world. It is locally named kudretnarı and papara in Turkey. *M. charantia* fruits are used as vegetable in various region of the world. Apart from their role in food consumption, a wide array of pharmacological activities of *M. charantia *fruits such as antihyperglycemic, antidiabetic, antiulcer, antifungal, protein synthesis inhibitory activity, anti-tumor and antioxidant effects have been reported. *M. charantia* fruits contain many bioactive chemicals as flavonoids, saponions, peptides, lectins, triterpenoids, phenolic compounds (13, 14).

In this study, free radical scavenging and antidiabetic activities of TAMC are reported and investigated by considering harvesting times. In addition, the potential correlation among the α-amylase and α-glucosidase enzyme inhibitions, free radical scavenging activities and anthocyanin contents were analyzed. The TAMC could be used as a possible food supplement and for treatment of some health problems as cancer, diabetes mellitus, hypertension, virus inhibition and Alzheimer’s disease in pharmaceutical and medicinal industry.

## Experimental

*Chemicals*

Sodium chloride, ferric chloride, sodium hydroxide, sodium carbonate and sodium acetate were purchased from E. Merck (Darmstadt, Germany). Acarbose, anhydrous ethanol, anhydrous dichloromethane, anhydrous ethyl acetate, glacial acetic acid, 2, 2-diphenyl-1-picryl-hydrazyl (DPPH^•^), *p*-nitrophenyl-α-D-glucopyranoside, starch, α-amylase, α-glucosidase, *N, N*-dimethyl-*p*-phenylendiamine (DMPD^•+^), 2, 2ꞌ-azino-bis (3-ethylbenzthiazoline-6-sulfonic acid) diammonium salt (ABTS^•+^), 3, 5-dinitrosalicylic acid (DNS), butylated hydroxyanisole (BHA), rutin hydrate, trolox, potassium persulfate and potassium sodium tartrate tetra hydrate were purchased from Sigma (Sigma-Aldrich GmbH, Sternheim, Germany). All other chemicals were of analytical grade and obtained from either Sigma-Aldrich or Merck.

*Preparation* *of* *fruit* *materials* *and extraction of total anthocyanins*

*Momordica charantia *Linn, from Cucurbitaceae family, was purchased from public market, in August, September, October and November 2012 in Antalya. It was identified by botanist Dr. İlginç Kızılpınar Temizer, Giresun University, Vocational High School of Health Services, Department of Medical Services and Techniques. Then, fruits were left in drying oven at 40˚C. The dried *M. charantia* fruits were chopped into 7 mm of particles. After that, CH_3_COOH (1.0 %) was added onto the fruit materials (150 g) at a rate of 1: 15, which yielded to 2250 mL of solution. The extraction process has been continued during 2 h at a room temperature, using magnetic blender. Extract was filtered by the paper filter and the received solution was 1800 mL. Solution was treated with dichloromethane and ethyl acetate four times (250 mL x 4) for each, respectively. The remaining solution, approximately 1000 mL, was dried in the lyophilizator (Christ Alpha 1–2 LD Plus) at 10 μm Hg pressure at -50˚C. Finally, the residues were placed in a plastic flask and then kept at -30˚C until used.

*Determination of Antidiabetic Activity*

*Assay of α-Amylase Inhibition*

*In-vitro *α-amylase inhibition was analyzed by following the method of Bernfeld (15) with minor modifications. The starch solution (0.5 %) was obtained by boiling and stirring potato starch (0.25 g) in deionized water (50 mL) for 15 min. The α-amylase (EC 3.2.1.1) enzyme solution (0.5 unit/mL) was prepared by mixing α-amylase (0.001 g) in phosphate buffer solution (PBS) (100 mL, 20 mM, pH 6.9) containing 6.7 mM sodium chloride. TAMC samples (5–100 μg/mL) and acarbose were dissolved at various concentrations in PBS. The color reagent was a solution containing DNS (20 mL, 96 mM), sodium potassium tartrate (8 mL, 5.31 M) in 2.0 M sodium hydroxide and deionized water (12 mL). 1 mL of samples (TAMC or acarbose) and enzyme solution (1.0 mL) were mixed in a tube and incubated at 25˚C for 30 min. 1 mL of this mixture was added to starch solution (1.0 mL) and the tube incubated at 25˚C for 3 min. Then, the color reagent (1.0 mL) was added and the closed tube placed into an 85˚C water bath. After 15 min, the reaction mixture was removed from the water bath and cooled thereafter, diluted with distilled water (9.0 mL) and the absorbance was recorded at 540 nm using spectrophotometer (Optizen Pop UV / Vis Single Beam Spectrophotometer) and α-amylase inhibition activities were expressed as IC_50 _(the concentration required to inhibition of α-amylase activity by 50%). The IC_50_ values were determined by linear regression analysis using four different concentrations in triplicate and represent mean of the data. Individual blanks were prepared for correcting the background absorbance. In this case, the color reagent solution was added prior to the addition of starch solution and then the tube placed into the water bath. The other procedures were carried out as above. Controls were conducted in an identical fashion replacing TAMC with PBS (1.0 mL). Acarbose solution was used as positive control.

*Assay of α-Glucosidase Inhibition*

A previously described bioassay method with minor modifications was used for measurement α-glucosidase inhibition of samples (16). The enzyme solution is contained α-glucosidase (EC 3.2.1.20) (20 μL, 0.5 unit/mL) and PBS (120 μL, 0.1 M, pH 6.9). *p*-nitrophenyl-α-D-glucopyranoside (5.0mM) in the PBS was used as a substrate solution. TAMC samples and acarbose (5–100 μg/mL, 10 µL), dissolved at various concentrations in PBS, were mixed with enzyme solution and incubated during 15 min at 37˚C. Substrate solution (20 µL) was added and incubated during 15 min. The reaction was terminated by adding sodium carbonate solution (80 μL, 0.2 M) and absorbance was measured at 405 nm using spectrophotometer. The IC_50_ values of samples for the α-glucosidase inhibition activities were determined by linear regression analysis using four different concentrations in triplicate and represent mean of the data.

*Determination of Free Radical Scavenging Activities*

*DPPH* *Radical Scavenging Activity Assay*

The DPPH radical scavenging abilities of samples were performed according to method of Blois (17) with minor modifications. Serially diluted samples (200 µL) at the different concentrations (5-30 μg/mL) was added to DPPH^• ^solution (2.8 mL, 0.2 mM) in ethanol. The mixtures were shook forcefully and allowed to stand at room temperature in the dark during 30 min. Then, absorbance was recorded at 517 nm in a spectrophotometer. The results were expressed as SC_50 _(the concentration required for scavenging DPPH radical by 50%) by linear regression analysis.

*DMPD* ^•+^ *Radical Scavenging Activity Assay*

Principal of the assay is based on reduction of the purple-colored radical DMPD^•+ ^described by Fogliano *et al*. (18). DMPD^•+^ solutions (100 mM) was prepared in a deionized water. This solution (1 mL) was added to acetate buffer (100 mL, 0.1 M, pH 5.25) and the colored radical cation (DMPD^•+^) was obtained by adding 0.2 mL of a of ferric chloride solution (0.05 M) (the final concentration was 0.01 mM). This solution (225 μL) was directly transferred to the tube and its absorbance was measured at 505 nm (absorbance of control tube). Different concentrations of TAMC samples or standards (15 μL, 5 to 30 μg/mL) and DMPD^•+^ (210 μL) were added to all tubes. Then, all tubes were stirred and left to stand for 10 min. After this time, a decrease in absorbance was measured at 505 nm in a spectrophotometer (absorbance of samples or standards). The buffer solution was used as a blank sample. The results were expressed as SC_50_ by linear regression analysis using four different concentrations in triplicate and represent mean of the data.

*ABTS* ^•+^ *Radical Scavenging Activity Assay*

**Figure 1:**
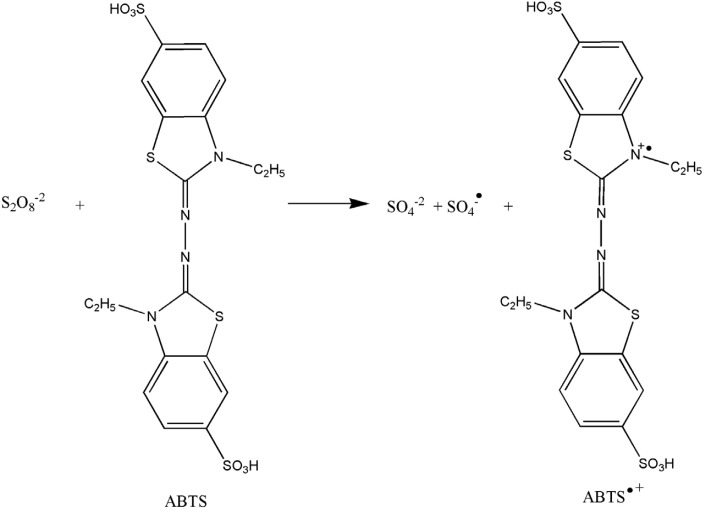
Generation of ABTS^•+ ^radical cation in the ABTS/K_2_S_2_O_8 _system

ABTS^•+ ^radical cation scavenging capacity of TAMC samples and standards was examined according to chemical methods described by Re *et al*. (19) with slight modification. This method is based on the ability of antioxidants to quench the long-lived ABTS^•+ ^radical cation, a blue/green chromophore with characteristic absorption at 734 nm, in comparison to that of BHA, rutin and trolox. Briefly, ABTS^•+^ radical cation was generated by a reaction of 2.0 mmol/L ABTS and 2.45 mmol/L potassium persulfate (Figure 1). The reaction mixture was allowed to stand in the dark for 16 h at room temperature and used within 2 days. Prior to assay, the ABTS^•+^ solution was diluted with PBS (0.1 M pH 7.4) to give an absorbance of 0.750 ± 0.020 at 734 nm in 1 cm cuvette and all the assays were performed by equilibrating at 30˚C temperature. Then, the diluted ABTS^•+^ solution (1.0 mL) was added to TAMC samples or standards (3.0 mL) solution in PBS at different concentrations (2.5–15 µg/mL). The percentage inhibition of ABTS^•+^ was calculated for each concentration relative to a blank absorbance. The results were expressed as SC_50_ by linear regression analysis using four different concentrations in triplicate and represent mean of the data.

*Determination of Total Anthocyanin Contents*

The total anthocyanin contents were carried out according to pH differential method described by Fuleki and Francis (20). The dried extracts (100 mg) were added to HCl (5.0 mL, 1.0 %) centrifuged at 3000 rpm for 10 min (MSE Mistral 2000, London, U.K.). Two supernatant tubes (0.2 mL) were prepared with buffer solutions having pH values of 1.0 and 4.5 respectively. Absorbance values were measured by using a spectrophotometer (Optizen Pop UV / Vis Single Beam Spectrophotometer) at 520 and 700 nm. Following buffer solutions were used as blank tubes in this experiment.

*pH 1.0 buffer (potassium chloride, 0.025M):* 1.86 g KCl was dissolved in 980 mL double-distilled water in a baker and the pH was adjusted to 1.0 ± 0.05 with HCl. The solution was transferred into 1 L volumetric flask and diluted to the volume with double-distilled water.

*pH 4.5 buffer (sodium acetate, 0.4 M.):* 54.43 g CH_3_COONa.3H_2_O was dissolved in 960 mL double-distilled water in a baker and the pH was adjusted 4.5 ± 0.05 with HCl. The solution was transferred into 1 L volumetric flask and diluted to the volume with double-distilled water.

Total anthocyanin contents in the extracts determined as mg/L of cyanidin-3-glucoside (cyd-3-glu) equivalent using the following equation:

Anthocyanin pigment (cyd-3-glu equivalent, mg/L)=A x MWcyd-3-glu x DF x 103ε × l

Equation 1

Where *A*: (*A*_520nm_ – *A*_700nm_) _pH 1.0_ – (*A*_520nm_ – *A*_700nm_) _pH 4.5_, MW_cyd-3-glu_ (molecular weight of cyanidin-3-glucoside): 449.2 g/mol, DF: dilution factor, *l*: path length in cm, ε = 26900 molar extinction coefficient for cyd-3-glu (L x mol^–1^ x cm^–1^) and 10^3^: factor for conversion from g to mg.

*Statistical analysis*

Experimental results were given as mean ± SD of the three parallel measurements. Analysis of variance was performed by ANOVA procedures. Significant differences between means were determined by Duncan’s Multiple Range tests. p-values of < 0.05 were regarded as significant and P-values of < 0.01 very significant. Both operations were done with SPSS 15.0 for windows.

## Results and Discussion

*Total Anthocyanin Contents*

Anthocyanins and other flavonoids are regarded as an important nutraceuticals mainly due to their antioxidant effects. Moreover, they have also been used to modulate the activity of a wide range of enzymes and cell receptors. Regular consumption of anthocyanins and other polyphenols in fruits, vegetables, wines, jams and preserves is associated with probable reduced risks of chronic diseases as cancer, cardiovascular diseases, virus inhibition and Alzheimer’s disease (1). Total anthocyanin contents in extracts found to be in a range from 24.48 ± 0.58 to 31.04 ± 1.22 mg/L as cyanidin 3-glucoside equivalents (Table 1). The phytochemicals such as phenolics and flavonoids are well known for their health benefits. The colorful anthocyanins are the most recognized biologically active flavonoids (30).

*α-Amylase Inhibition Activity*

Recently, natural sources of α-amylase inhibitor have received a lot of interest due to the side or mild effects as of synthetic enzyme inhibitors such as acarbose, metformin and orlistat and at the same time synthetic enzyme inhibitors can cause gastrointestinal distress (21). Certain plant phenolics have the ability to partially inhibit the activity of α-amylase enzyme and hence they demonstrated therapeutic benefits such as hypoglycemic effect and are therefore useful in dietary management of type II diabetes (22). The α-amylase inhibitory activities were studied in concentrations range from 5 to 100 μg/mL. α-amylase inhibitory activity of TAMC samples was compared with standard acarbose with an IC_50_ value of 93.07 ± 1.49 μg/mL. The IC_50_ value of TAMC-A, TAMC-S, TAMC-O and TAMC-N were found to be 56.86 ± 1.12, 63.81 ± 0.86, 66.18 ± 1.34, 71.62 ± 1.01 μg/mL, respectively (*p *< 0.05). According to obtained results, TAMC showed appreciable α-amylase inhibitory effects when compared with acarbose. The author also suggests that *M. charantia *has high antidiabetic potential due to the total anthocyanins in it.

*α-Glucosidase Inhibition Activity*

α-glucosidase inhibitors such as acarbose, miglitol and voglibose are widely used in the treatment of patients with type II diabetes. α-glucosidase inhibitors delay the absorption of carbohydrates from the small intestine and thus they have a lowering effect on postprandial blood glucose and insulin levels (23). Earlier studies have reported that the retardation α-glucosidase enzyme by inhibitors would be one of the most effective ways to control type II diabetes (22, 24). α-glucosidase inhibitory activity of extracts was compared with standard acarbose with an IC_50_ value of 77.25 ± 1.20 μg/mL. The IC_50_ values of TAMC-A, TAMC-S, TAMC-O and TAMC-N were found to be 88.19 ± 0.74, 97.99 ± 1.40, 100.55 ± 1.73, 107.68 ± 0.98 μg/mL, respectively (*p *< 0.05). These results show that total athocyanins of *M. charantia *may be potential α-glucosidase inhibitor for diabetic disorder.

*DPPH* ^•^ *Radical Scavenging Activity*

**Figure 2:**
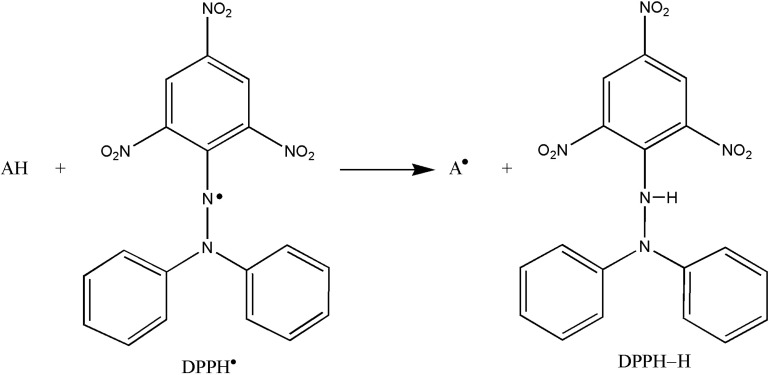
Reaction scheme between antioxidant (AH) and DPPH^• ^radical

The effect of antioxidants on DPPH^•^ radical scavenging is due to their hydrogen or electron donating abilities. DPPH^•^, a stable free radical, is transformed to stable diamagnetic molecule by receiving an electron or a hydrogen radical (Figure 2). In its radical form, DPPH^•^ absorbs at 517 nm, but this absorbance value decreases in the presence of an antioxidant or a radical species due to the reaction between antioxidant molecules and radical. It is visually noticeable as a change in color from purple to yellow (5, 25). As a consequence, DPPH^•^ is usually used as a substrate to evaluate the antioxidative activity of antioxidants (26). The SC_50_ values (µg/mL) of TAMC samples and standards on the DPPH^• ^increased in that order: TAMC-S > (2.55 ± 0.08) > TAMC-O (3.13 ± 0.02) > TAMC-A (4.93 ± 0.24) > TAMC-N (7.39 ± 0.10) > BHA (8.94 ± 0.21) > rutin (17.47 ± 0.17) > trolox (25.74 ± 0.46) (*p *< 0.05) (Table 1).

*DMPD* ^•+^ *Radical* *Scavenging Activity*

**Figure 3:**
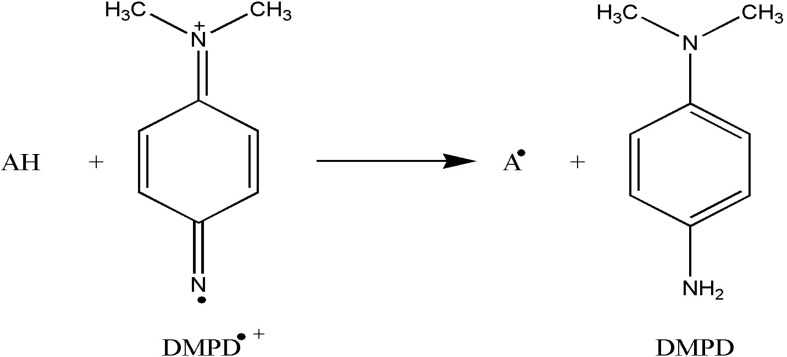
Reaction scheme between antioxidant (AH) and DMPD^•+ ^radical cation

Dark color of DMPD^•+^ radical cation solution becomes lighter and absorbance of solution decreases in the presence of an antioxidant compound. The DMPD^•+ ^radical cation solution shows a maximum absorbance at 505 nm. Antioxidant compounds which are hydrogen donors to DMPD^•+^ quench the color of DMPD^•+^ solution (27). Figure 3 illustrates a reaction between antioxidant and DMPD^•+^. SC_50 _value for TAMC-A, TAMC-S, TAMC-O and TAMC-N were 5.04 ± 0.41, 2.68 ± 0.09, 5.04 ± 0.02, 5.41 ± 0.04 μg/mL, respectively. These values were found as 10.69 ± 0.09, 14.83 ± 0.34 and 29.96 ± 0.68 μg/mL for rutin, BHA and trolox, respectively (*p *< 0.01) (Table 1).

**Table 1:** Radical scavenging activities and total anthocyanin contents of *M. charantia *fruit.

	**DPPH** ^a^	**DMPD** ^a^	**ABTS** ^a^	**Total Anthocyanin Contents** ^b^
TAMC-A	4.93 ± 0.24	5.04 ± 0.41	9.31 ± 0.16	27.66 ± 0.91
TAMC-S	2.55 ± 0.08	2.68 ± 0.09	8.19 ± 0.09	31.04 ± 1.22
TAMC-O	3.13 ± 0.02	5.04 ± 0.02	8.56 ± 0.02	29.47 ± 0.37
TAMC-N	7.39 ± 0.10	5.41 ± 0.04	9.62 ± 0.15	24.48 ± 0.58
BHA	8.94 ± 0.21	14.83 ± 0.34	8.47 ± 0.19	X
RUT	17.47 ± 0.17	10.69 ± 0.09	15.68 ± 0.39	X
TRO	25.74 ± 0.46	29.96 ± 0.68	4.18 ± 0.08	X

TAMC-A (Total Anthocyanins of *M. charantia*-August) TAMC-S (Total Anthocyanins of *M. charantia*-September) TAMC-O (Total Anthocyanins of *M. charantia*-October) TAMC-N (Total Anthocyanins of *M. charantia*-November) BHA (Butylated hydroxyanisole), RUT: Rutin, TRO: Trolox. (p > 0.05).

a SC50 values (µg/mL)

b mg/L cyanidin-3-glucoside equivalent

*ABTS* ^•+^ *Radical* *Scavenging Activity*

Bleaching of a preformed solution of the blue-green radical cation ABTS^•+^ has been extensively used to evaluate the antioxidant capacity of complex mixtures and individual compounds. The reaction of the preformed radical with free-radical scavengers can be easily monitored by following the decay of the sample absorbance at 734 nm. The ABTS^•+ ^radical cation can be prepared employing different oxidants. Results obtained using a potassium persulfate as oxidant show that the presence of peroxodisulfate increases the rate of ABTS^•+ ^autobleaching in a concentration-dependent manner. ABTS^•+ ^radical cation was generated in the ABTS/K_2_S_2_O_8_ system (28). The reaction scheme can be denotable as follow:

S_2_O_8_^-2^ + ABTS → SO_4_^-2^ + SO_4_^•-^ + ABTS^•+^                     (1)

SO_4_^•-^ +ABTS →SO_4_^-2^ + ABTS^•+^                    (2)

S_2_O_8_^-2^ + 2ABTS →2SO_4_^-2^ + 2ABTS^•+^

**Figure 4:**
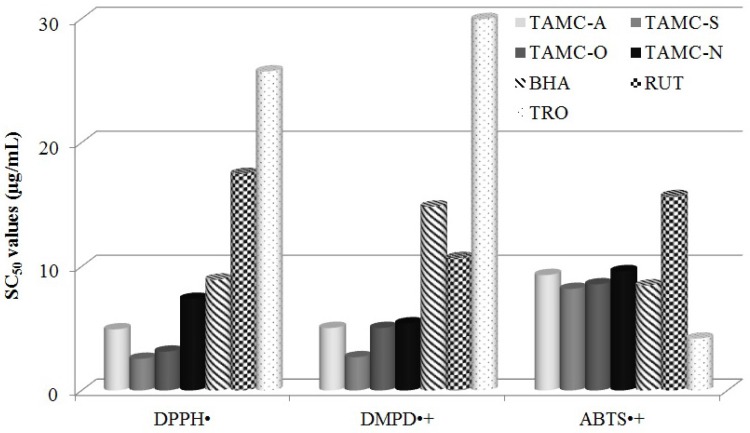
DPPH^•^, ABTS^•+^ and DMPD^•+^ radical scavenging activities of total anthocyanins from *M. charantia *fruits (TAMC) and standards. TAMC-A (Total Anthocyanins of *M. charantia*-August) TAMC-S (Total Anthocyanins of *M. charantia*-September) TAMC-O (Total Anthocyanins of *M. charantia*-October) TAMC-N (Total Anthocyanins of *M. charantia*-November) BHA (Butylated Hydroxyanisole), RUT: Rutin, TRO: Trolox

Generation of the ABTS^•+ ^radical cation forms the basis on one of the spectrophotometric methods that have been applied to the measurement of the total antioxidant activity of pure substances, solutions, aqueous mixtures and beverages (29). For radical scavenging activities, the SC_50_ values of the TAMC samples and standards are reported in Figure 4. The results show that ABTS^•+ ^radical cation scavenging activities of the TAMC samples are very important as well as standards such as BHA and rutin. SC_50 _values of TAMC samples and standards on the ABTS^•+ ^radical cation decreased in that order: trolox (4.18 ± 0.08) < TAMC-S (8.19 ± 0.09) < BHA (8.47 ± 0.19) < TAMC-O (8.56 ± 0.02) < TAMC-A (9.31 ± 0.16) < TAMC-N (9.62 ± 0.15) < rutin (15.68 ± 0.39) (*p *< 0.01) (Table 1).

The correlation coefficient between DPPH radical scavenging activity and total anthocyanin contents was statistically significant (*R* = 0.984, *p *< 0.05). Similarly, those between anthocyanin contents and DMPD radical scavenging activity (*R* = 0.951, *p *< 0.05), ABTS radical scavenging activity (*R* = 0.988, *p *< 0.05) were significant. Additionally, the correlation between total anthocyanin contents and α-amylase inhibition activity (*R* = 0.962, *p *< 0.05), α-glucosidase inhibition activity (*R* = 0.976, *p *< 0.05) were significant.

## Conclusions

History showed that medicinal plants have been used in traditional healing around the world for a long time for the treatment of many diseases *i.e*. diabetes, asthma, eczema, premenstrual syndrome, rheumatoid arthritis, migraine, menopausal symptoms, *etc*. and can be used for maintaining general health. A lot of medicinal plants in the literature have hypoglycemic and other beneficial properties. When they are correctly used, medicinal plants are considered safer than conventional medications. People are greatly concerned about the efficacy and side effects of many synthetic drugs and hence they choose herbal medicines for providing a safe and natural alternative treatment for many health problems. In fact, the herbs are always the alternative medicine and primary source. The advantages of using medicinal plants are numerous. Otherwise, they typically have fewer side effects and may be safer to use over time. *M. charantia* fruits may be useful for the treatment of antidiabetic by reduction the α-amylase and α-glucosidase enzymes activities due to the presence of anthocyanin contents in it. In addition to this TAMC samples demonstrated very effective scavenging activities depend on the DPPH^•^, DMPD^•+^ and ABTS^•+^ radicals but these properties change by depending on harvest times. When these activities compared with the synthetic antioxidants, they are very important for regulation of oxidative stress owing to theirs side effects. By this way, the obtained results in this study show that total anthocyanins of *M. charantia* fruits can be used as easy accessible source of natural antidiabetics and antioxidants as a possible food supplement or in a pharmaceutical and medical industry. For this reason, it could be performed on the isolation and characterization of the anthocyanin compounds from TAMC and to evaluate its *in-vivo* effects in next works.

## References

[1] Andersen ØM, Markham KR (2006). Flavonoids: Chemistry, Biochemistry, and Applications. CRC Press, Broken Sound Parkway NW.

[2] Glade MJ (1997). Food, nutrition, and the prevention of cancer: A global perspective. American Institute for Cancer Research/World Cancer Research Fund, American Institute for Cancer Research 1997. Nutrition.

[3] Middleton E, Kandaswami C, Theoharides TC (2000). The effects of plant flavonoids on mammalian cells: implications for inflammation, heart disease, and cancer. Pharmacol Rev.

[4] Khanna RS, Pandey A, Negi R, Pande D, Karki K, Khanna HD, Khanna S (2011). Characterization and evaluation of antioxidant activity. Indian J Res.

[5] Güder A, Korkmaz H (2012). Evaluation of in-vitro antioxidant properties of hydroalcoholic solution extracts Urtica dioica L, Malva neglecta Wallr and their mixture. Iran J Pharm Res.

[6] Korekar G, Stobdan T, Singh H, Chaurasia OP, Singh SB (2011). Phenolic content and antioxidant capacity of various solvent extracts from sea buckthorn (Hippophae rhamnoides L) fruit pulp, seeds, leaves and stem bark. Acta Alimentaria.

[7] Lachance PA, Nakat Z, Jeong WS (2001). Antioxidants: an integrative approach. Nutrition.

[8] Güder A, Korkmaz H (2012). Investigation of antioxidant activity and total anthocyanins from blackberry (Rubus hirtus Waldst and Kit) and cherry laurel (Laurocerasus officinalis Roem). Asian J Chem.

[9] Huxley RR, Neil HAW (2003). The relationship between dietary flavonol intake and coronary heart diseasemortality: a meta-analysis of prospective cohort studies. Eur J Clin Nutr.

[10] Güder A, Engin MS, Yolcu M, Gür M (2014). Effect of processing temperature on the chemical composition and antioxidant activity of Vaccinium arctostaphylos fruit and their jam. J Food Process Pres.

[11] Kavishankar GB, Lakshmidevi N, Murthy MS, Prakash HS, Niranjana SR (2011). Diabetes and medicinal plants-A review. Int J Pharm Biomed Sci.

[12] Chai TT, Elamparuthi S, Yong AL, Quah Y, Ong HC, Wong FC (2013). Antibacterial, anti-glucosidase, and antioxidant activities of selected highland ferns of Malaysia. Bot Stud.

[13] Baytop T (2000). Therapy with Medicinal Plants in Turkey (Past and Present). Nobel Tip Press, Istanbul.

[14] Shan B, Xie JH, Zhu JH, Peng Y (2012). Ethanol modified supercritical carbon dioxide extraction of flavonoids from Momordica charantia L. and its antioxidant activity. Food Bioprod Process.

[15] Bernfeld P, Colowick SP, Kaplan NO (1955). Amylase, α and β. Methods in Enzymology.

[16] McCue P, Kwon YI, Shetty K (2005). Anti-amylase, anti-glucosidase and anti-angiotensin I-converting enzyme potential of selected foods. J Food Biochem.

[17] Blois MS (1958). Antioxidant determinations by the use of a stable free radical. Nature.

[18] Fogliano V, Verde V, Randazzo G, Ritieni A (1999). Method for measuring antioxidant activity and its application to monitoring the antioxidant capacity of wines. J Agric Food Chem.

[19] Re R, Pellegrini N, Proteggente A, Pannala A, Yang M, Rice-Evans C (1999). Antioxidant activity applying an improved ABTS radical cation decolorization assay. Free Rad Biol Med.

[20] Fuleki T, Francis FJ (1968). Determination of total anthocyanin and degradation index for cranberry juice. J Food Sci.

[21] Randhir R, Kwon Y, Shetty K (2008). Effect of thermal processing on phenolics, antioxidant activity and health-relevant functionality of select grain sprouts and seedling. Innov Food Sci Emerg Tech.

[22] Chethan S, Sreerama YN, Malleshi NG (2008). Mode of inhibition of finger millet malt amylases by the millet phenolics. Food Chem.

[23] Van De Laar FA, Lucassen PL, Akkermans RP, Van De Lisdonk EH, Rutten GE, Van Weel C (2005). α-Glucosidase inhibitors for patients with type 2 diabetes. Diabetes Care.

[24] Kunyanga CN, Imungi JK, Okoth MW, Biesalski HK, Vadivel V (2012). Total phenolic content, antioxidant and antidiabetic properties of methanolic extract of raw and traditionally processed Kenyan indigenous food ingredients. LWT-Food Sci Technol.

[25] Sreena KP, Poongothai A, Soundariya SV, Srirekha G, Santhi R, Annapoorani S (2011). Evaluation of in-vitro free radical scavenging efficacy of different organic extracts of Morinda tinctoria leaves. Int J Pharm Sci.

[26] Ancerewicz J, Migliavacca E, Carrrupt PA, Testa B, Bree F, Zini R, Tillement JP, Labidelle S, Guyot D, Chauvet-Monges AM, Crevat A, Le Ridant A (1998). Structure-property relationships of trimetazidine derivatives and model compounds as potential antioxidants. Free Rad Bio Med.

[27] Koksal E, Bursal E, Dikici E, Tozoglu F, Gulcin I (2011). Antioxidant activity of Melissa officinalis leaves. J Med Plant Res.

[28] Gülçin I (2010). Antioxidant properties of resveratrol: A structure–activity insight. Innov Food Sci Emerg Tech.

[29] Miller HE, Rigelhof F, Marquart L, Prakash A, Kanter M (2000). Antioxidant content of whole grain breakfast cereals, fruits and vegetables. J Am Coll Nutr.

[30] Kumar S, Gautam S, Sharma A (2013). Identification of antimutagenic properties of anthocyanins and other polyphenols from Rose (Rosa centifolia) petals and tea. J Food Sci.

